# Deep learning-based chemical shift-artifact correction of ZTE MRI for enhanced bone depiction of the lumbar spine

**DOI:** 10.1007/s00256-026-05290-4

**Published:** 2026-07-06

**Authors:** Carina Obermüller, Ulf Bach, Fabio Zecca, Franziska Heidt, Maelene Lohezic, Sagar Mandava, Jose de Arcos Rodriguez, Florian Wiesinger, Roman Guggenberger, Egon Burian, Jonas Kroschke, Falko Ensle

**Affiliations:** 1 Diagnostic and Interventional Radiology, University Hospital Zurich, University of Zurich, Zurich, Switzerland; 2 Department of Medical Imaging, Oncopole Claudius Regaud - IUCT Oncopole, Toulouse, France; 3 GE HealthCare, Zurich, Switzerland; 4 GE HealthCare, Atlanta, GA USA; 5 GE HealthCare, Valencia, Spain; 6 GE HealthCare, Munich, Germany; 7 Department of Radiology and Nuclear Medicine, Cantonal Hospital Winterthur, University of Zurich, Zurich, Switzerland

**Keywords:** Magnetic resonance imaging, Lumbar vertebrae, Deep learning, Image reconstruction, Artifacts, Image interpretation, computer-assisted

## Abstract

**Rationale and objectives:**

Zero echo time (ZTE) is an advanced MRI technique providing CT-like images of mineralized tissues. This study evaluates the impact of deep learning (DL)–based reconstruction with chemical shift correction (DLCSC) on osseous depiction in ZTE MRI of the lumbar spine, compared to standard DL and non-DL reconstruction.

**Methods:**

This retrospective, single-center study included 38 patients undergoing 3 T ZTE MRI of the lumbar spine. K-space data were reconstructed using three methods: non-DL, standard DL, and a prototype DLCSC algorithm. Quantitative image sharpness, signal-to-noise ratio (SNR), and contrast-to-noise ratio (CNR) were analyzed using repeated-measures ANOVA. Two radiologists independently rated pathology-related criteria (*n* = 22) and bone depiction quality (*n* = 38) on a 4-point scale. Ordinal data were analyzed using the Friedman test, and inter-reader agreement was assessed with weighted Cohen’s kappa.

**Results:**

DLCSC images yielded quantitatively sharper images compared to non-DL (*p* = 0.010) and standard DL (*p* < 0.001). SNR and CNR were significantly higher in DLCSC and DL than in non-DL (*p* < 0.001). In the qualitative assessment, mean scores for all criteria of pathologies and bone depiction quality improved significantly from non-DL and DL to DLCSC (*p* < 0.001). There was no evidence of differences in classification of pathologies (*p* > 0.05). Inter-reader agreement ranged from substantial to almost perfect (*κ* = 0.867–0.901).

**Conclusion:**

DLCSC image reconstruction of ZTE MRI can improve bone depiction of the lumbar spine compared to standard DL and non-DL reconstruction.

**Supplementary Information:**

The online version contains supplementary material available at https://doi.org/10.1007/s00256-026-05290-4.

## Introduction

Zero echo time (ZTE) is an advanced magnetic resonance imaging (MRI) technique that can capture signal from ultrashort-T2 tissues (e.g., bone, teeth, lung), capable of providing CT-like images of mineralized tissues [1, 2]. ZTE can be seamlessly appended to standard MRI protocols for improving the diagnostic confidence during the assessment of both soft tissues and bone [3]. This is particularly desirable in spinal imaging, where additional CT is often required (e.g., preoperative planning) [4]. With its inherent three-dimensional capabilities, ZTE has demonstrated potential as a radiation-free alternative for preoperative planning and intraoperative navigation [5]. Deep learning (DL)–based reconstruction has further enhanced bone depiction of ZTE imaging by mitigating its signal-to-noise ratio (SNR) limitations via denoising and edge sharpening. Encouraging results have been obtained in various body regions, including the shoulder, knee, and cervical spine [6–8]. However, in the body trunk, ZTE imaging of the spine remains challenging due to additional chemical shift artifacts, which lead to blurring or destructive interference at tissue interfaces with consequent degradation of bone delineation [9, 10]. Such artifacts, which originate from radial k-space filling of the ZTE sequence, are more pronounced in the spine due to the surrounding soft tissue bulk, particularly at higher field strengths (e.g., 3 Tesla (T)) [11]. Existing conventional strategies to reduce these artifacts come at unfavorable/undesirable costs, such as poor SNR efficiency when scanning at high bandwidths [12]. Due to these limitations, previous studies have mostly resorted to other MR techniques for CT-like images of the lumbar spine, mainly spoiled gradient echo (SPGR) sequences [13, 14]. However, relative to ZTE, gradient echo sequences provide inferior suppression of soft tissues neighboring the bone, which could compromise cortical bone detail [15, 16]. Recently, a novel method has been introduced to mitigate the effects of chemical shift artifacts in ZTE MRI using a deep learning–based approach. Combining DL-based denoising and edge sharpening with chemical shift-artifact correction (DLCSC) could enable ZTE as the optimal technique for detailed osseous assessment of the spine, within clinically reasonable scan times [17, 18]. The purpose of this study was to assess the impact of DLCSC on bone depiction in ZTE MRI of the lumbar spine at 3 T compared to conventional (non-DL) reconstructions, as well as to assess its added value relative to DL reconstruction without chemical shift correction.

## Materials and methods

### Study design

**Fig. 1:**
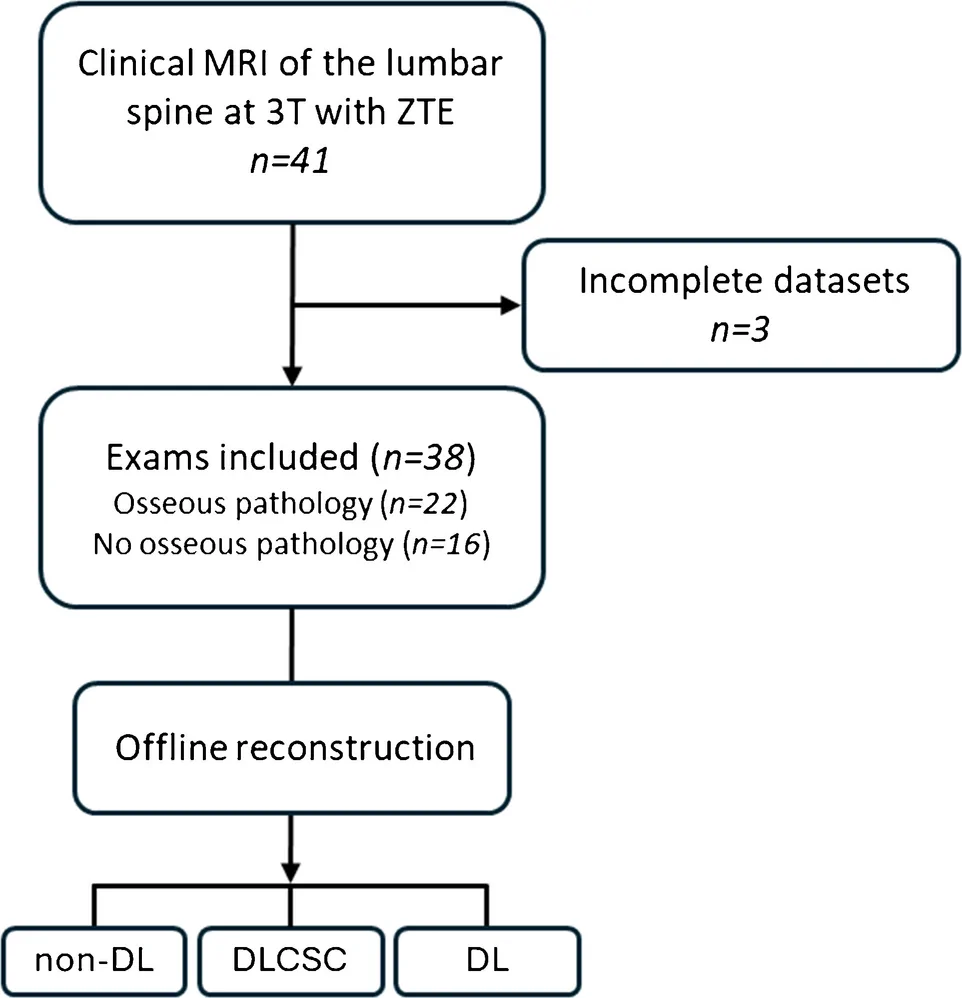
Flow diagram of patient inclusion. DL, deep learning; CSC, chemical shift-artifact correction

Between December 2024 and August 2025, patients referred for a clinically indicated MRI exam of the lumbar spine at 3 T were considered for study inclusion. Exclusion criteria were (1) any incomplete ZTE sequence dataset impeding DL reconstruction and (2) patient age < 18 years. The project has been approved by the local ethics commission and the methodology described by the STROBE Checklist for observational studies was followed (Supplemental Table 1). A flow chart with the detailed inclusion process is shown in Fig. 1.

### Image acquisition

All scans were acquired on a 3 T MRI System (SIGNA™ Premier, GE HealthCare, Waukesha, WI, USA) with a 60-channel posterior array coil. As part of the institution’s standard MR protocol, an isotropic ZTE pulse sequence was obtained in the sagittal plane. The acquisition parameters were selected to be representative of clinical protocols with a balance between scan time and diagnostic yield: echo time ≅ 0; repetition time = 444, flip angle = 1°; pixel bandwidth = 244 Hz; receiver bandwidth = 62.5 kHz; spokes per segment = 384; number of excitations = 8; field of view = 26 cm; slice thickness = 1.3 mm; acquisition matrix = 512 × 512; scan time = 2 min 33 s.

### Image reconstruction

The raw k-space data of the ZTE acquisitions were retrieved from the MR scanner and anonymized. Three sets of images were reconstructed offline and provided for analysis, with the images gray-scale inverted for CT-like visualization. The first set was generated using a conventional reconstruction pipeline, referred to as “non-DL.” The second set was processed using a previously described DL model for noise reduction and sharpening of ZTE data, referred to as “DL” [6, 7]. The third set was processed using the enhanced deep learning model with the specific chemical shift correction, referred to as “DLCSC.” There was full randomization of all datasets for quantitative and qualitative analysis.

Both DL algorithms were provided by the vendor as prototypes. The models are extensions of the AIR™ Recon DL method [19], specifically adapted to the ZTE acquisition and to perform additional chemical shift correction in the case of DLCSC. The network was trained in a supervised manner using pairs of images. Low-bandwidth ZTE images, which exhibit chemical shift artifacts and noise, served as the input. High-quality, double-bandwidth images from the same subjects were used as the ground truth or target. To ensure the model’s robustness to variations in anatomy, field strength, and noise levels, extensive data augmentation was performed during training, including image rotations, flips, and manipulations of image intensity and phase. To further improve robustness, additional image noise was also added. The trained network is designed to remove chemical shift artifacts and noise while recovering fine anatomical details. The DLCSC model processes raw k-space data from the non-DL pipeline to produce the final ZTE images.

### Quantitative image analysis

Quantitative comparison between reconstruction methods was conducted only in the subgroup without osseous abnormalities (*n* = 16). The subgroup with pathologies was not used for quantitative analysis to avoid any potential distortion of measurements.

#### Edge sharpness

**Fig. 2:**
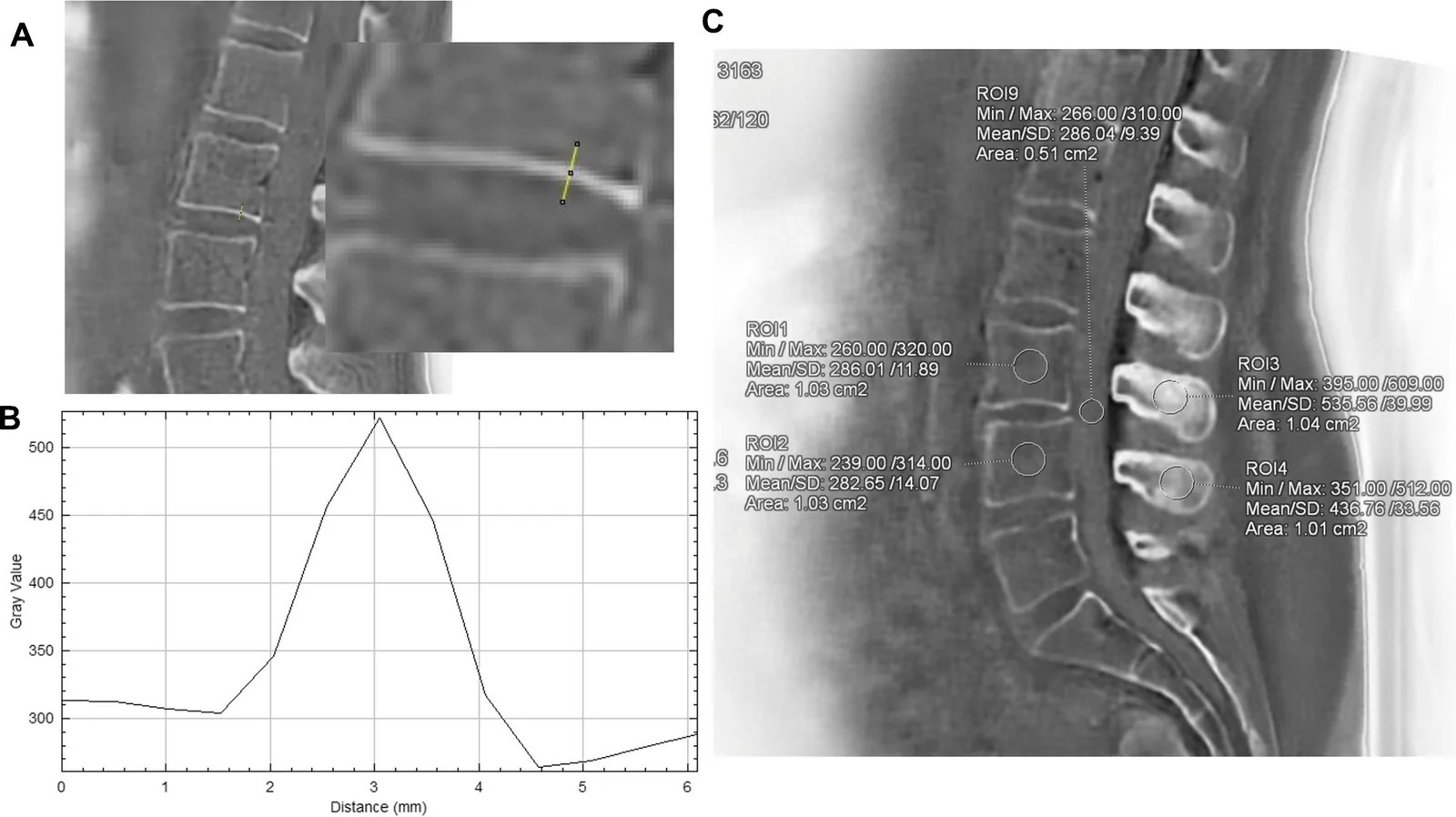
**A**–**C** Sample images of quantitative analyses. Measurement of edge sharpness (yellow line in **A**) with line profile (**B**) drawn through the inferior vertebral endplate of L3, and region-of-interest measurements (**C**) for SNR and CNR calculations

Edge sharpness (*Š*) of cortical bone was assessed quantitatively based on a previously described method [20]. Measurements were performed by a fellowship-trained musculoskeletal radiologist with 6 years of experience. A sampling line was drawn orthogonally to the inferior endplate of L2, L3, and L4 at its posterior third on the median sagittal plane (Fig. 2A, B). The same lines were overlaid on the non-DL, DL, and DLCSC series on the corresponding median slices. The signal intensity (SI) data obtained from the sampling lines were extracted to a datasheet and plotted. For each resulting SI curve, being ΔSI the SI interval of the steepest segment of the curve, Δ*x* the corresponding distance, and S̅I̅ the corresponding average SI, *Š* was calculated as in Eq. 1. The resulting values from L2, L3, and L4 were then averaged into a series-specific value.

1 $$\check {S}=\frac{(\Delta\mathrm{S}\mathrm{I}/\Delta x)}{\underline{\mathrm{S}\mathrm{I}}}$$

#### SNR and CNR

Analysis of apparent (a)SNR and contrast-to-noise ratio (aCNR) was performed by a radiology resident with 3 years of experience, under the supervision of a board-certified radiologist with 7 years of experience. On a single midline sagittal image, regions-of-interest (ROIs) were manually placed in the following locations: at the center of the vertebral body and spinous process of L3 and L4, respectively, for trabecular bone measurements (10 mm^2^); in the center of the intervertebral disc L3/4 and in the spinal canal at the intervertebral level L3/L4 (5 mm^2^) (Fig. 2C). On a parasagittal image, ROIs were placed in the cortical bone of the pars interarticularis and the adjacent paravertebral muscle at the level L4/5 (5 mm^2^). Mean and standard deviation (SD) values of SI were computed for each ROI. The mean of all trabecular bone measurements served as the bone signal value for the calculations to obtain estimates of noise from various locations inside the anatomy. The pars interarticularis was used to calculate cortical bone aCNR, as the cortical bone in this location was thick enough to allow a representative ROI placement. aSNR and aCNR were calculated according to previously published formulas [7, 8], whereby aCNR was calculated using the spinal canal for the trabecular bone aCNR calculations and the intervertebral disc or paravertebral muscle for the cortical bone aCNR calculations:

$$aSNR=\frac{SI\left(bone\right)}{SD\left(bone\right)}$$

$$aCNR=\frac{SI\left(bone\right)-SI\left(spinal\;canal\right)}{SD\left(bone\right)}$$

$$aCNR=\frac{SI\left(bone\right)-SI\left(intervertebral\;disc\right)}{SI\left(bone\right)}$$

$$aCNR=\frac{SI\left(bone\right)-SI\left(paravertebral\;muscle\right)}{SI\left(bone\right)}$$

### Qualitative image analysis

Qualitative image analysis was performed by a board-certified radiologist with 7 years and a radiology resident with 4 years of experience in musculoskeletal MRI, respectively. All anonymized ZTE image sets were evaluated independently in a random order on PACS using diagnostic quality monitors. Raters were blinded to the reconstruction method, and to any clinical information. Prior to independent grading, both readers attended a training session led by a senior musculoskeletal radiologist with more than 15 years of experience, to establish consensus on the grading criteria. The separate training set consisted of five cases not included in the study analysis.

Bone depiction quality was subjectively evaluated by grading the following parameters: (1) delineation of cortical and trabecular bone, (2) noise, (3) artifacts, and (4) diagnostic image quality. Supplemental Figure 1 provides an illustrative overview of the gradings for bone depiction. For the subset with osseous pathologies (*n* = 22), the following additional parameters were scored: (1) type of pathology, (2) conspicuity of pathology, (3) diagnostic confidence, and (4) subjective impact of chemical shift artifacts on depiction of pathologies for the raters. Grading criteria are demonstrated in Table 1.

**Table 1:** Overview of the 4-point scoring system used for assessing bone depiction and pathologies

Score	Delineation of cortical bone	Delineation of trabecular bone	Noise	Artifacts	Diagnostic image quality	Type of pathology	Conspicuity of pathology	Diagnostic confidence	Impact of chemical shift artifacts
0	Poor (cortical borders obscured)	Poor (trabecular structure obscured)	Non- acceptable	None	Poor/non-diagnostic	Degenerative changes	Poor/non-diagnostic	Poor/non-diagnostic	None
1	Moderate (cortical blurring)	Moderate (trabecular blurring)	Strong	Artifact obscuring only non-bony structures	Moderate	Fracture/vertebral height reduction	Moderate	Moderate	Partially, diagnosis/severity grading of diagnosis not affected
2	Good (most cortical borders sharply delineated)	Good (most trabecular structure delineated)	Fair	Artifact obscuring bony structures, diagnostic confidence not impacted	Good	Spondylolysis	Good	Good	Substantially, diagnosis/severity grading of diagnosis affected
3	Excellent (all cortical borders sharply delineated)	Excellent (trabecular structure fully delineated)	Minimal	Artifact obscuring bony structures, diagnostic confidence impacted	Excellent	Spondylolysthesis	Excellent	Excellent	-

### Statistical analysis

Normality of data was assessed using the Shapiro-Wilk test applied to the pairwise within-subject differences for each pair of reconstruction conditions (see supplementary Table 2). Accordingly, descriptive statistics (means, standard deviations, medians, quartiles, and ranges) were reported for each condition. Quantitative image analysis metrics, including edge sharpness, SNR, and CNR, were analyzed using repeated-measures analysis of variance (ANOVA) to compare differences across the three reconstruction methods. Post hoc pairwise comparisons with Bonferroni correction were performed to identify specific differences. Qualitative analyses were performed using the Friedman test with post hoc Bonferroni corrections. Bonferroni correction was applied within each metric’s three pairwise reconstruction comparisons. Each patient contributed exactly one summarized value per reconstruction condition. Inter-reader reliability was assessed using quadratic-weighted Cohen’s kappa (*κ*). Degree of agreement was considered poor (*κ* < 0), slight (*κ* = 0.00–0.20), fair (*κ* = 0.21–0.40), moderate (*κ* = 0.41–0.60), substantial (*κ* = 0.61–0.80), or near-perfect (*κ* = 0.81–1.00). All statistical analyses were implemented using Python 3.12. *p*-values below 0.05 were deemed to indicate statistical significance.

## Results

In total, 38 exams of 38 patients were included for the final analysis. Patients presented for MRI with the following clinical history (*n*): radiculopathy (*n* = 17), trauma (*n* = 12), unspecific lower back pain (*n* = 8), and inflammatory disease (*n* = 1). A summary of participant characteristics is shown in Table 2. The included exams were divided into two subgroups with and without osseous pathologies, classified by a senior musculoskeletal radiologist with more than 15 years of experience in the role of a super partes expert.

**Table 2:** Patient demographics and subgroups

Characteristics	No osseous pathologies	Osseous pathologies
Examinations (*n*)	16	22
Age (years, median (IQR))	43 (40–50)	63 (51–69)
Males/females (*n*, %)	Male: 3 (19%) Female: 13 (81%)	Male: 8 (36%) Female: 14 (64%)
Osseous pathology (*n*)	-	Degenerative changes (12) Fracture/vertebral height reduction (3) Spondylolysis (4) Spondylolysthesis (3)

### Quantitative image analysis

#### Edge sharpness

The quantitative analysis of image sharpness showed a statistically significant main effect of the reconstruction technique on image sharpness (*F*(2, 30) = 15.619, *p* < 0.001; confirmed by non-parametric sensitivity analysis: Friedman *Q* = 15.129, *p* < 0.001). DLCSC reconstructions (mean sharpness = 0.388 ± 0.193) were significantly sharper than both the non-DL (mean = 0.269 ± 0.086; *p* = 0.010, mean difference = 0.119, 95% CI [0.046, 0.192], *T* = 3.472, Hedges *g* = 0.775) and DL reconstruction (mean = 0.247 ± 0.106; *p* = 0.001, mean difference = −0.141, 95% CI [−0.205, −0.077], *T* = −4.722, Hedges *g* = −0.885). There was no evidence of a difference in sharpness between non-DL and DL reconstructions (*p* = 0.252, mean difference = −0.023, 95% CI [−0.048, 0.003], *T* = −1.850, Hedges *g* = −0.226). Details can be found in supplementary Tables 3 and 4.

#### SNR and CNR

SNR and CNR calculations of trabecular bone showed a significant main effect across the three reconstruction techniques (SNR: *F*(2, 30) = 30.248, *p* < 0.001, confirmed by non-parametric sensitivity analysis: Friedman *Q* = 24.500, *p* < 0.001; CNR: *F*(2, 30) = 15.501, *p* < 0.001, confirmed by non-parametric sensitivity analysis: Friedman *Q* = 20.375, *p* < 0.001). Both DLCSC and DL techniques demonstrated a substantial increase in mean SNR compared to the non-DL technique. Specifically, non-DL reported a mean SNR of 16.070 ± 1.715, while DL reported 28.609 ± 8.129, and DLCSC reported 27.888 ± 6.700. Post hoc analysis confirmed that both DL (*p* < 0.001, mean difference = 12.539, 95% CI [8.803, 16.275], *T* = 7.154, Hedges *g* = 2.081) and DLCSC (*p* < 0.001, mean difference = 11.818, 95% CI [8.566, 15.071], *T* = 7.745, Hedges *g* = 2.356) resulted in significantly higher SNR compared to non-DL. No evidence of a difference in SNR was observed between DL and DLCSC (*p* = 1.000, mean difference = 0.721, 95% CI [−3.767, 5.208], *T* = 0.342, Hedges *g* = 0.094). Concerning trabecular bone CNR, DLCSC demonstrated the highest mean (2.994 ± 1.890), followed by DL (2.277 ± 1.652) and non-DL (1.023 ± 0.524). Post hoc analysis showed that both DL (*p* = 0.006, mean difference = 1.253, 95% CI [0.532, 1.975], *T* = 3.703, Hedges *g* = 0.997) and DLCSC (*p* < 0.001, mean difference 1.970, 95% CI [1.19, 2.751], *T* = 5.380, Hedges *g* = 1.385) had significantly higher trabecular bone CNR than non-DL. The difference in trabecular bone CNR between DLCSC and DL was not statistically significant (*p* = 0.213, mean difference = −0.717, 95% CI [−1.504, 0.07], *T* = −1.942, Hedges *g* = −0.394). No evidence of differences was found in CNR of cortical bone relative to the intervertebral disc (non-DL 3.296 ± 1.482, DL 3.863 ± 2.419, DLCSC 3.744 ± 1.480, *F*(2, 30) = 1.004, *p* = 0.3784, confirmed by non-parametric sensitivity analysis: Friedman *Q* = 3.500, *p* = 0.174) or to the paravertebral muscles (non-DL 3.019 ± 1.317, DL 3.595 ± 2.173, DLCSC 3.382 ± 1.378, *F*(2, 30) = 1.081, *p* = 0.3522, confirmed by non-parametric sensitivity analysis: Friedman *Q* = 4.500, *p* = 0.105). Details can be found in supplementary Table 3.

### Qualitative image analysis

The qualitative assessment demonstrated a significant difference across the three reconstructions for all evaluated image quality criteria, including diagnostic image quality, cortical and trabecular bone delineation, noise, and artifacts (see Tables 3 and 4). Mean scores (Table 3) progressively increased from non-DL to DL to DLCSC. Specifically, DLCSC received the highest scores for diagnostic image quality (mean ± SD non-DL 0.961 ± 0.243, DL 1.711 ± 0.413, DLCSC 2.382 ± 0.538), cortical bone delineation (mean ± SD non-DL 0.947 ± 0.517, DL 1.842 ± 0.421, DLCSC 2.605 ± 0.509), trabecular bone delineation (mean ± SD non-DL 1.079 ± 0.487, DL 1.605 ± 0.522, DLCSC 2.289 ± 0.488), and noise (mean ± SD non-DL 1.000 ± 0.558, DL 1.803 ± 0.443, DLCSC 2.579 ± 0.395), while demonstrating the lowest (best) scores for artifacts (mean ± SD non-DL 2.000 ± 0.797, DL 1.684 ± 0.512, DLCSC 1.276 ± 0.446).

**Table 3:** Comparison of qualitative gradings of ZTE images reconstructed with non-deep learning (non-DL), DL, and DL with chemical shift-artifact correction (DLCSC) methods. Mean of two readers with standard deviation and inter-reader agreement with Cohen’s kappa are given

Variable	Non-DL (mean ± SD)	DL (mean ± SD)	DLCSC (mean ± SD)	Cohen’s kappa	Cohen’s kappa 95% CI
Delineation of cortical bone	0.947 ± 0.517	1.842 ± 0.421	2.605 ± 0.509	0.880	[0.819, 0.930]
Delineation of trabecular bone	1.079 ± 0.487	1.605 ± 0.522	2.289 ± 0.488	0.805	[0.737, 0.870]
Noise	1.000 ± 0.558	1.803 ± 0.443	2.579 ± 0.395	0.828	[0.752, 0.888]
Artifacts	2.000 ± 0.797	1.684 ± 0.512	1.276 ± 0.446	0.783	[0.658, 0.877]
Diagnostic image quality	0.961 ± 0.243	1.711 ± 0.413	2.382 ± 0.538	0.901	[0.833, 0.952]
Type of pathology	1.909 ± 0.984	2.091 ± 1.065	2.318 ± 1.041	0.803	[0.604, 0.938]
Conspicuity of pathology	1.182 ± 0.501	1.773 ± 0.528	2.477 ± 0.545	0.789	[0.636, 0.897]
Diagnostic confidence	1.136 ± 0.441	2.227 ± 0.685	2.568 ± 0.470	0.901	[0.824, 0.961]
Impact of chemical shift artifacts	1.023 ± 0.607	0.705 ± 0.454	0.409 ± 0.479	0.867	[0.761, 0.955]

**Table 4:** Test statistics for each comparison of qualitative ratings of ZTE images reconstructed with non-deep learning (non-DL), DL, and DL with chemical shift-artifact correction (DLCSC) methods

Variable	Comparison	Mean difference	95% CI	*T* statistic	*p*-value (uncorrected)	*p*-value (Bonferroni)	Hedges *g*
Delineation of cortical bone	DL vs. non-DL	0.895	[0.793, 0.997]	17.734	0.000	< 0.001	1.879
Delineation of cortical bone	DL vs. DLCSC	−0.763	[−0.882, −0.644]	−12.969	0.000	< 0.001	−1.618
Delineation of cortical bone	Non-DL vs. DLCSC	−1.658	[−1.796, −1.520]	−24.285	0.000	< 0.001	−3.199
Delineation of trabecular bone	DL vs. non-DL	0.526	[0.347, 0.705]	5.957	0.000	< 0.001	1.032
Delineation of trabecular bone	DL vs. DLCSC	−0.684	[−0.861, −0.507]	−7.839	0.000	< 0.001	−1.340
Delineation of trabecular bone	Non-DL vs. DLCSC	−1.211	[−1.445, −0.976]	−10.464	0.000	< 0.001	−2.458
Noise	DL vs. non-DL	0.803	[0.588, 1.017]	7.579	0.000	< 0.001	1.577
Noise	DL vs. DLCSC	−0.776	[−0.937, −0.616]	−9.786	0.000	< 0.001	−1.831
Noise	Non-DL vs. DLCSC	−1.579	[−1.752, −1.406]	−18.481	0.000	< 0.001	−3.236
Artifacts	DL vs. non-DL	−0.316	[−0.576, −0.055]	−2.458	0.019	0.056	−0.467
Artifacts	DL vs. DLCSC	0.408	[0.217, 0.598]	4.339	0.000	< 0.001	0.841
Artifacts	Non-DL vs. DLCSC	0.724	[0.460, 0.988]	5.557	0.000	< 0.001	1.109
Diagnostic image quality	DL vs. non-DL	0.750	[0.625, 0.875]	12.130	0.000	< 0.001	2.190
Diagnostic image quality	DL vs. DLCSC	−0.671	[−0.810, −0.532]	−9.766	0.000	< 0.001	−1.384
Diagnostic image quality	Non-DL vs. DLCSC	−1.421	[−1.572, −1.270]	−19.124	0.000	< 0.001	−3.368
Type of pathology	DL vs. non-DL	0.182	[−0.237, 0.601]	0.902	0.377	1.000	0.174
Type of pathology	DL vs. DLCSC	−0.227	[−0.499, 0.044]	−1.742	0.096	0.288	−0.212
Type of pathology	Non-DL vs. DLCSC	−0.409	[−0.806, −0.012]	−2.144	0.044	0.132	−0.397
Conspicuity of pathology	DL vs. non-DL	0.591	[0.379, 0.803]	5.786	0.000	< 0.001	1.127
Conspicuity of pathology	DL vs. DLCSC	−0.705	[−0.984, −0.425]	−5.247	0.000	< 0.001	−1.289
Conspicuity of pathology	Non-DL vs. DLCSC	−1.295	[−1.557, −1.034]	−10.285	0.000	< 0.001	−2.430
Diagnostic confidence	DL vs. non-DL	1.091	[0.738, 1.444]	6.425	0.000	< 0.001	1.859
Diagnostic confidence	DL vs. DLCSC	−0.341	[−0.600, −0.081]	−2.732	0.012	0.037	−0.570
Diagnostic confidence	Non-DL vs. DLCSC	−1.432	[−1.708, −1.156]	−10.782	0.000	< 0.001	−3.083
Impact of chemical shift artifacts	DL vs. non-DL	−0.318	[−0.551, −0.086]	−2.846	0.010	0.029	−0.583
Impact of chemical shift artifacts	DL vs. DLCSC	0.295	[0.094, 0.497]	3.052	0.006	0.018	0.622
Impact of chemical shift artifacts	Non-DL vs. DLCSC	0.614	[0.398, 0.829]	5.919	0.000	< 0.001	1.102

**Fig. 3:**
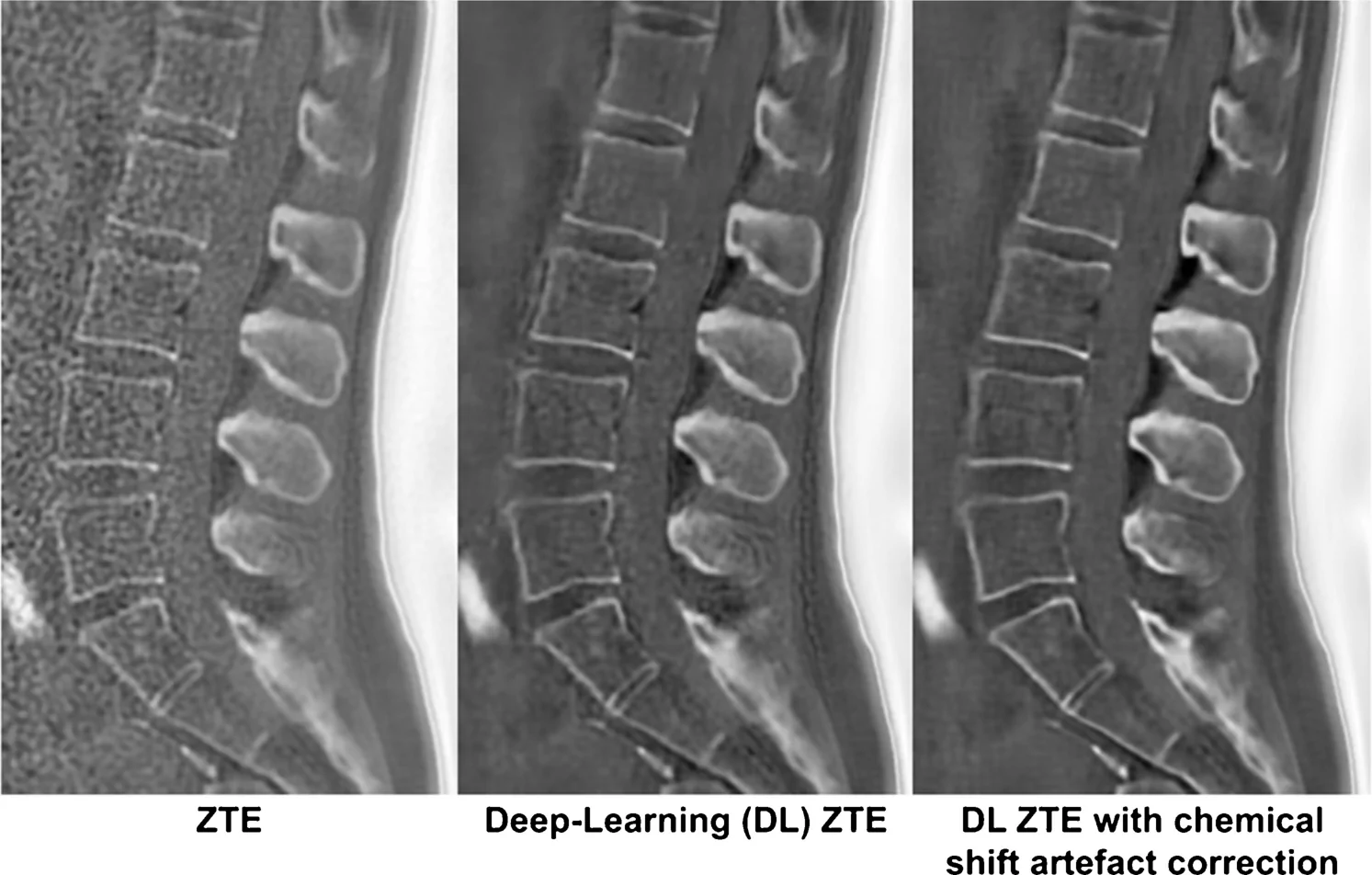
Example of a 32-year-old male patient demonstrating bone delineation in sagittal ZTE images with deep learning (DL)–based chemical shift artifact correction (DLCSC), DL without CSC (DL), and non-DL reconstructions. The non-DL image displays marked noise, which is largely removed by the DL reconstruction. The DLCSC image demonstrates additional removal of chemical shift artifacts, resulting in sharper delineation of osseous contours. No bony abnormalities were noted

**Fig. 4:**
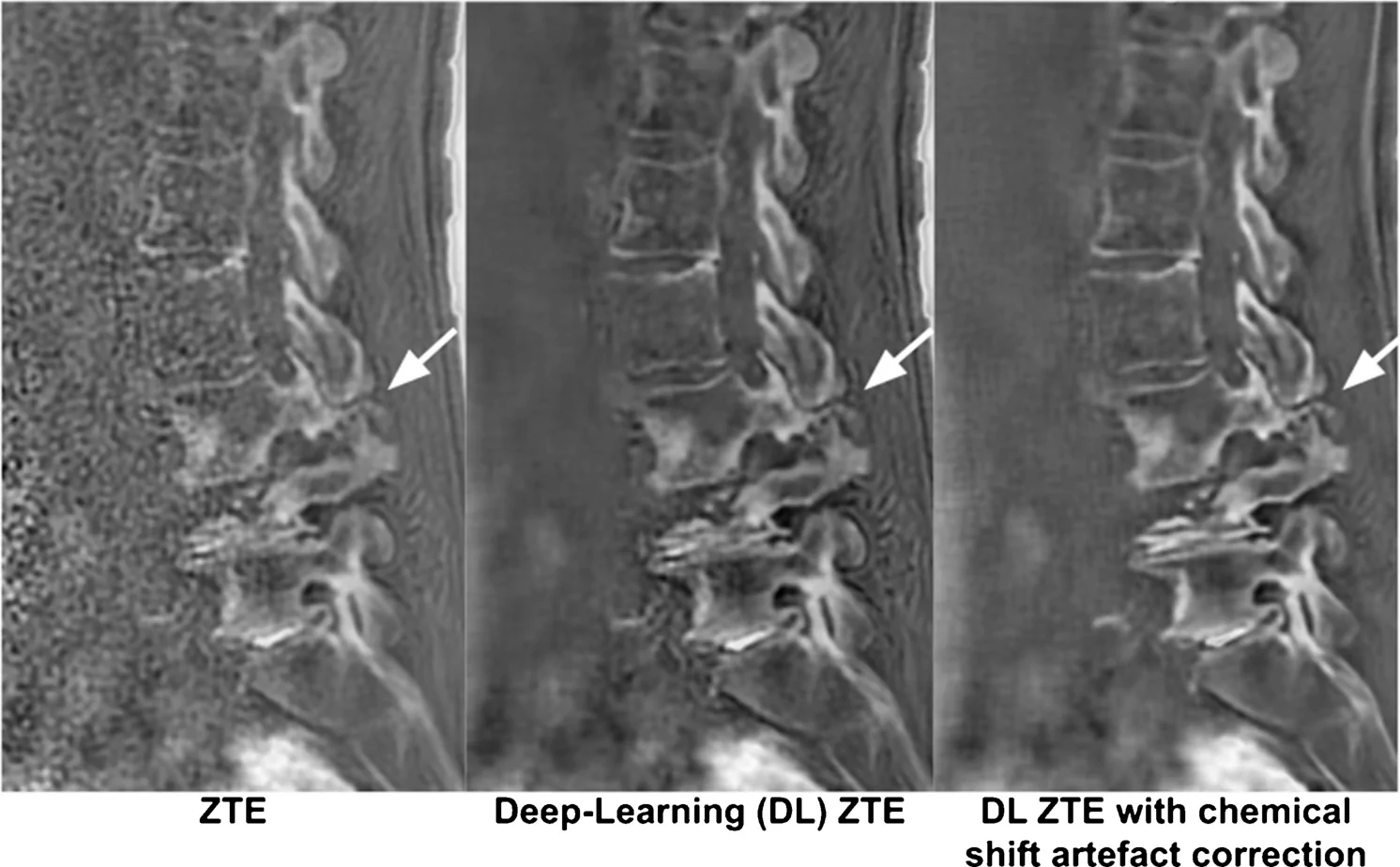
A 53-year-old woman with lumbar scoliosis. Sagittal ZTE images demonstrate a left-sided pars defect (spondylolysis) of L3 (arrow). The ZTE image (non-DL) displays marked noise and chemical shift artifacts with blurring of the osseous structures. Deep learning (DL)–based ZTE reconstruction removes image noise and improves sharpness, although with persisting chemical shift artifacts. DL ZTE reconstruction with chemical shift-artifact correction (DLCSC) demonstrates additional removal of chemical shift artifacts, resulting in higher conspicuity of the pars defect

**Fig. 5:**
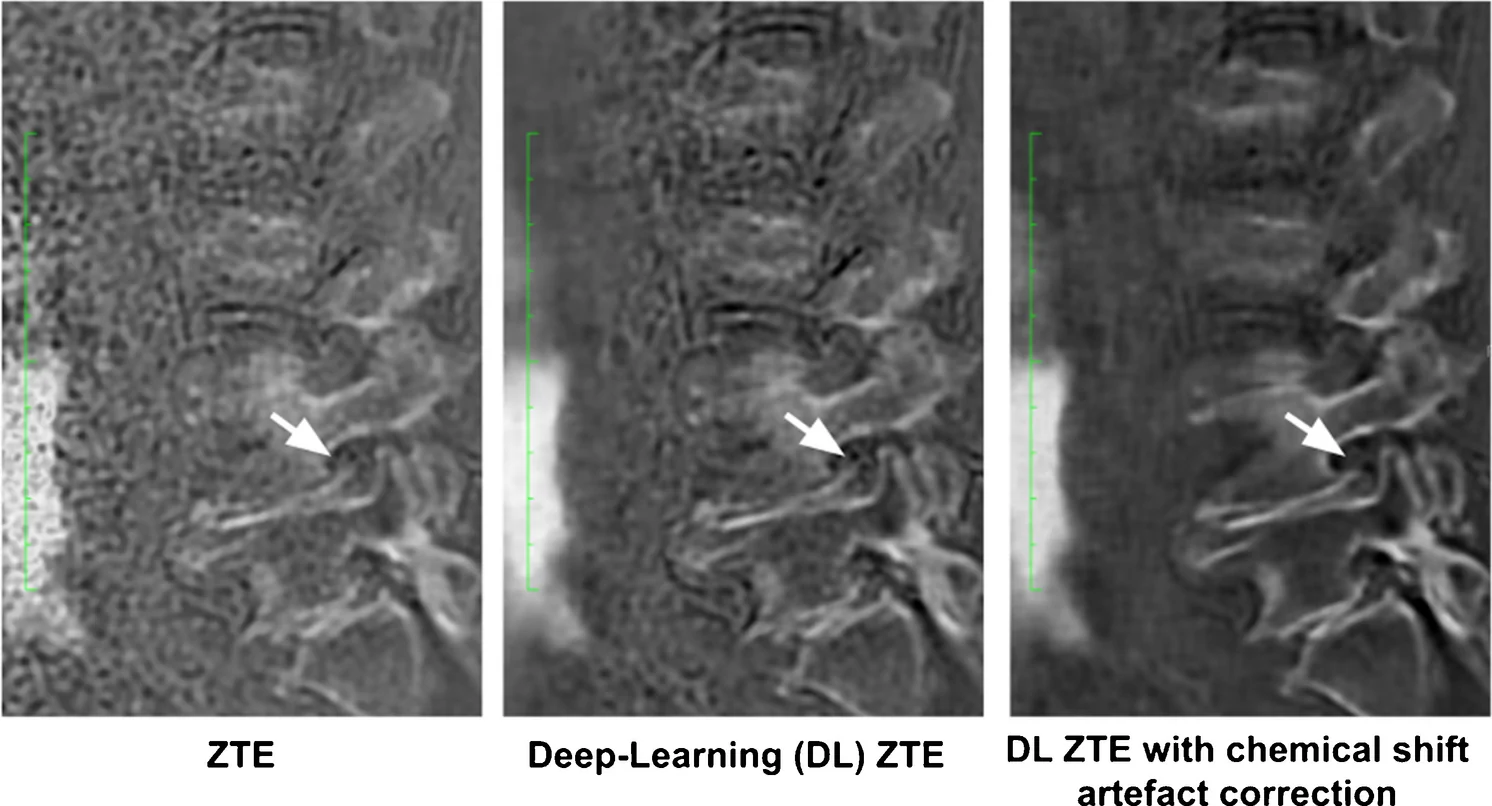
A 76-year-old woman with lumbar scoliosis. Sagittal ZTE images demonstrate an osseous spur extending into the neuroforamen of L4/5 (arrow). The ZTE image (non-DL) displays marked noise and chemical shift artifacts with blurring of the osseous spur. Deep learning (DL)–based ZTE reconstruction removes image noise and improves sharpness, although with persisting chemical shift artifacts. DL ZTE reconstruction with chemical shift-artifact correction (DLCSC) demonstrates additional removal of chemical shift artifacts, resulting in higher conspicuity of the osseous spur

**Fig. 6:**
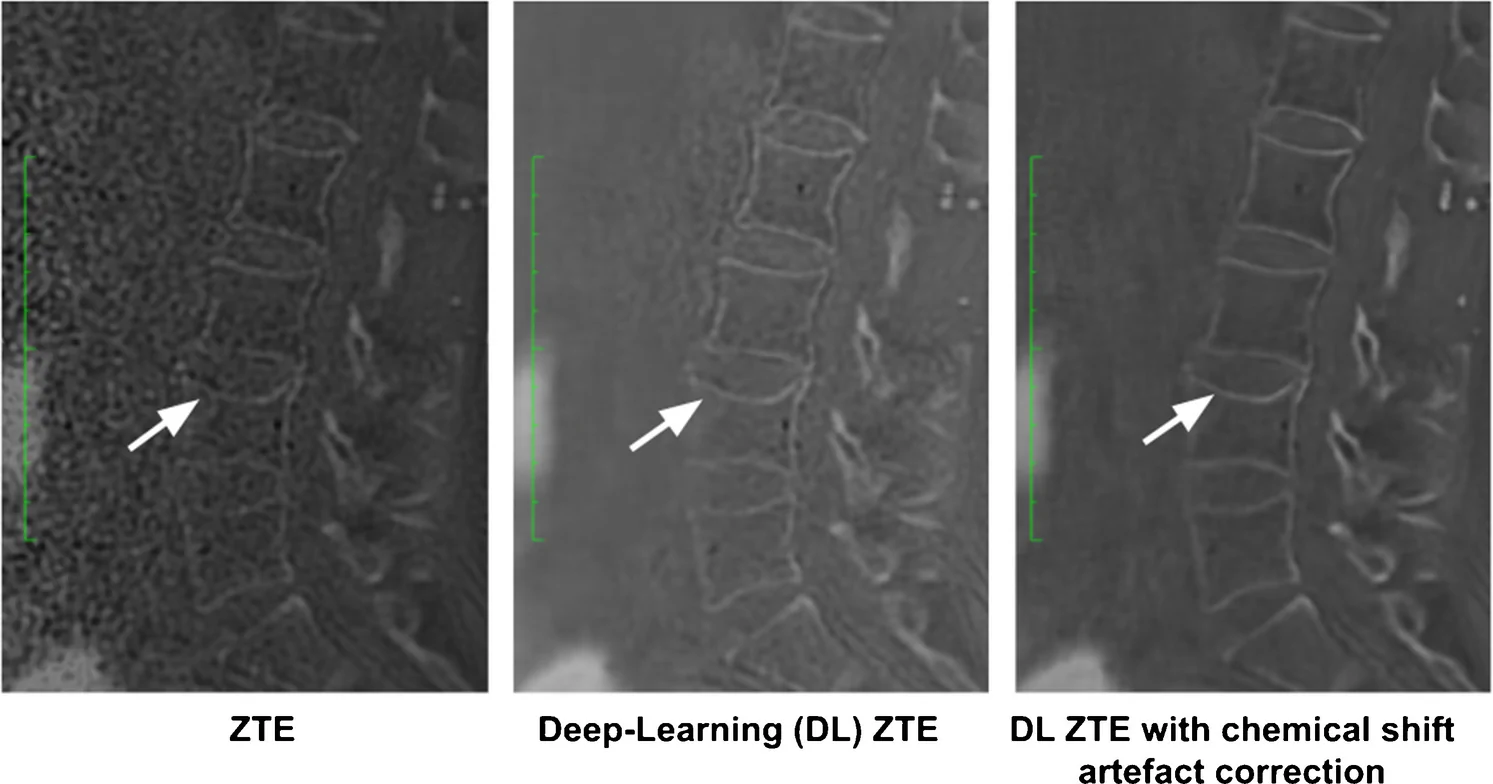
A 71-year-old woman with an endplate fracture. Sagittal ZTE images demonstrate a decreased height of the upper L4 vertebrae endplate (arrow). The ZTE image (non-DL) displays marked noise and chemical shift artifacts with blurring of the cortical border. Deep learning (DL)–based ZTE reconstruction removes image noise and improves sharpness, although with persisting chemical shift artifacts at the cortical bone. DL ZTE reconstruction with chemical shift artifact correction (DLCSC) demonstrates additional removal of chemical shift artifact adjacent to the cortical bone, resulting in higher conspicuity of the cortical bone of the endplate

Similarly, in the assessment of pathologies, there were statistically significant improvements in pathology conspicuity (mean ± SD non-DL 1.182 ± 0.501, DL 1.773 ± 0.528, DLCSC 2.477 ± 0.545), reduction of artifacts impacting bone depiction (mean ± SD non-DL 2.000 ± 0.797, DL 1.684 ± 0.512, DLCSC 1.276 ± 0.446), and diagnostic confidence (mean ± SD non-DL 1.136 ± 0.441, DL 2.227 ± 0.685, DLCSC 2.568 ± 0.470). No evidence of a difference was found among the reconstructions regarding the classification of pathology type. Details of the statistical test results can be found in Table 4. Figures 3, 4, 5, and 6 show examples of improved bone delineation in a case without pathology (Fig. 3), in a case of spondylolysis (Fig. 4), and in a case of neuroforaminal bony spur (Fig. 5) and a fracture (Fig. 6).

### Inter-reader agreement

The inter-reader agreement (Table 3) for the qualitative assessment was substantial to almost perfect across all categories. For pathology assessment, *κ* ranged from 0.789 (substantial) for conspicuity to 0.901 (almost perfect) for diagnostic confidence. In the bone depiction domain, agreement ranged from *κ* = 0.783 (substantial) for artifacts to *κ* = 0.901 (almost perfect) for diagnostic quality.

## Discussion

In this study, we assessed the utility of a novel DL-based reconstruction method with chemical shift-artifact correction (DLCSC) to enhance bone visualization in the lumbar spine using ZTE MRI at 3 T in the lumbar spine. Qualitative and quantitative results suggested that DLCSC significantly improves delineation of osseous structures, compared to DL without CSC and non-DL reconstructions. Along with enhanced image quality, DLCSC demonstrated improved subjective conspicuity of osseous pathologies and higher diagnostic confidence for the observers.

Accurate depiction of osseous findings in the lumbar spine is clinically relevant for assessing various pathologies, such as traumatic injuries or stenosing degenerative disease [21, 22]. However, conventional MRI often lacks the required osseous detail in clinical practice, resulting in referrals for additional CT acquisition. Implementing ZTE sequences as an adjunct to the routine spine protocol could enable MRI as a more comprehensive diagnostic tool [17, 18]. Recently, emerging DL algorithms for ZTE image reconstruction have further optimized the diagnostic efficacy of the technique by mitigating its SNR limitations with denoising and sharpening [23, 24]. However, chemical shift (CS) artifacts remain challenging to address without undesirable tradeoffs, such as image blurring or additional data acquisition [10, 12]. The center-out radial spoke used in ZTE data acquisition leads to phase accumulation in the off-resonant fat component, manifesting as high-frequency artifacts at tissue interfaces. These CS artifacts can occur in proximity to the bone-soft tissue interface, potentially compromising osseous assessment.

In the current work, DLCSC demonstrated significantly improved edge sharpness of cortical bone as measured by line profiling and compared to DL reconstructions. This indicates that CS artifacts affect bone depiction and contribute to blurring, even in denoised and sharpened images with the “standard” DL reconstruction. Quantitative analysis of aSNR and aCNR showed no evidence of differences between DL and DLCSC, further corroborating the notion that the additional improvement in qualitative bone delineation of DLCSC can be attributed to artifact reduction. In the subgroup with osseous pathologies, both observers noted a subjectively reduced impact of CS artifacts on bone depiction in the DLCSC images relative to DL and non-DL. Interestingly, the senior observer also noted a reduced impact of CS artifacts in the DL relative to non-DL images, while the resident observer did not note significant differences. The denoising and sharpening properties of the “standard” DL reconstruction without CSC led to sharper delineation of bone interfaces that could also be visually interpreted as a result of reduced CS artifacts.

The novel DLCSC model is an extension of the “standard” DL algorithm, specifically trained to remove CS artifacts and noise, while recovering fine details. A strength of the DLCSC method is its wide generalizability to any anatomy, field strength, undersampling rates, and noise levels. To our knowledge, this is the first clinical study exploring a DL-based approach to mitigate CS artifacts in the spine. The lumbar spine represents a desirable anatomy for this application, as the large osseous surface area together with surrounding soft tissue bulk predisposes to CS artifacts. Additionally, the abdominal girth can introduce substantial image noise in the anterior spine sections, particularly in obese patients, owing to increased distance from the receiver coil. The DLCSC method can mitigate both of these drawbacks simultaneously, removing noise and CS artifacts, without penalties in SNR efficiency. Our findings are in line with the currently only published study applying the DLCSC reconstruction to ZTE MRI, that recently reported enhanced quantitative and qualitative bone assessment of the temporomandibular joint [25].

Due to prior limitations, ZTE imaging has been relatively underutilized in the lumbar spine compared to other anatomies. Instead, research on lumbar applications for bone MRI has mainly focused on SPGR sequences, such as the detection of pars defects or in preoperative planning [13, 14]. While SPGR generally offers higher SNR and cortical bone to soft tissue contrast, ZTE is more robust to motion and provides flatter contrast of soft tissue [26, 27]. Employing DLCSC for optimal bone depiction could enable ZTE as a viable alternative also in the lumbar spine. The investigated ZTE sequence was acquired in a scan time of 2:30 min, which seems clinically feasible to implement in a routine MRI protocol of the spine.

Our study has several limitations. First, the retrospective design and the relatively small patient cohort from a single center may limit the generalizability of our findings, especially the small sample size used for the quantitative analysis, which may limit the statistical robustness of the ANOVA results. Furthermore, the subgroup of patients with osseous pathologies was heterogeneous, and certain conditions, such as fractures, were underrepresented. Consequently, the study is underpowered to draw definitive conclusions regarding the diagnostic performance of the reconstruction algorithm for specific types of spinal pathology. Second, the qualitative assessment was inherently subjective, and blinding was likely not fully effective, as visual image characteristics between the non-DL and both DL reconstructions were distinctive and recognizable to the observers. This limitation could introduce expectation bias for the raters. However, inter-reader agreement was substantial, and differences in imaging features between DLCSC and DL reconstructions were more subtle. Further, the qualitative results were supported by the quantitative analysis by two measurement methods. Third, we did not compare the ZTE findings against an external reference standard, such as CT, to definitively assess diagnostic accuracy for specific pathologies. Future studies should assess the impact of DLCSC reconstruction on the diagnostic performance of ZTE imaging, compared to reference standard CT.

In conclusion, our findings demonstrate that CS artifacts and image noise can be effectively mitigated by the DLCSC reconstruction method, improving bone depiction of the lumbar spine. While diagnostic accuracy and confidence were not compared to the reference standard of CT, this study focused on enhancing image quality to enable clinical utility of ZTE.

## Supplementary Information

Below is the link to the electronic supplementary material.ESM 1Supplementary Material 1 (DOCX 465 KB)

## Data Availability

Data is available upon reasonable request from the authors.

## Abbreviations

ANOVA: Analysis of variance
CNR: Contrast-to-noise ratio
DL: Deep learning
DLCSC: Deep learning with chemical shift correction
MRI: Magnetic resonance imaging
ROI: Region-of-interest
SD: Standard deviation
SI: Signal intensity
SNR: Signal-to-noise ratio
SPGR: Spoiled gradient echo
T: Tesla
ZTE: Zero echo time

## References

[1] Fujisaki A, Tsukamoto J, Narimatsu H, Hayashida Y, Todoroki Y, Hirano N, et al. Zero echo time magnetic resonance imaging; techniques and clinical utility in musculoskeletal system. J Magn Reson Imaging. 2024;59:32–42. 37288953 10.1002/jmri.28843

[2] Yan T-C, Yu S-W, Liu L, Xu J-H, Wang S-T, Li N, et al. Oxygen-enhanced ZTE-MRI for pulmonary nodule assessment: a comparative study with CT. Acad Radiol. 2025;32:4903–12. 40348712 10.1016/j.acra.2025.04.036

[3] Bharadwaj UU, Coy A, Motamedi D, Sun D, Joseph GB, Krug R, et al. CT-like MRI: a qualitative assessment of ZTE sequences for knee osseous abnormalities. Skeletal Radiol. 2022;51:1585–94. 35088162 10.1007/s00256-021-03987-2 PMC9198000

[4] Grimme J. CT of the head and spine. Acad Radiol. 2002;9:1071–2.

[5] Altorfer FCS, Burkhard MD, Kelly MJ, Avrumova F, Sneag DB, Chazen JL, et al. Robot-assisted lumbar pedicle screw placement based on 3D magnetic resonance imaging. Global Spine J. 2025;15:1243–50. 38324511 10.1177/21925682241232328 PMC11571642

[6] Ensle F, Kaniewska M, Lohezic M, Guggenberger R. Enhanced bone assessment of the shoulder using zero-echo time MRI with deep-learning image reconstruction. Skeletal Radiol. 2024;53:2597–606. 38658419 10.1007/s00256-024-04690-8 PMC11493801

[7] Ensle F, Abel F, Lohezic M, Obermüller C, Guggenberger R. Deep learning reconstruction for optimized bone assessment in zero echo time MR imaging of the knee. Eur J Radiol. 2024;179:111663. 39142010 10.1016/j.ejrad.2024.111663

[8] Kaniewska M, Zecca F, Obermüller C, Ensle F, Deininger-Czermak E, Lohezic M, et al. Deep learning reconstruction of zero-echo time sequences to improve visualization of osseous structures and associated pathologies in MRI of cervical spine. Insights Imaging. 2025;16:29. 39881081 10.1186/s13244-025-01902-0 PMC11780046

[9] Aydıngöz Ü, Yıldız AE, Ergen FB. Zero echo time musculoskeletal MRI: technique, optimization, applications, and pitfalls. Radiographics. 2022;42:1398–414. 35904982 10.1148/rg.220029

[10] Engström M, McKinnon G, Cozzini C, Wiesinger F. In-phase zero TE musculoskeletal imaging. Magn Reson Med. 2020;83:195–202. 31429994 10.1002/mrm.27928

[11] Teixeira PAG, Kessler H, Morbée L, Douis N, Boubaker F, Gillet R, et al. Mineralized tissue visualization with MRI: practical insights and recommendations for optimized clinical applications. Diagn Interv Imaging. 2025;106:147–56. 39667997 10.1016/j.diii.2024.11.001

[12] Weiger M, Pruessmann KP. Short-T MRI: principles and recent advances. Prog Nucl Magn Reson Spectrosc. 2019;114–115:237–70. 31779882 10.1016/j.pnmrs.2019.07.001

[13] Altorfer FCS, Yan JJ, Abel F, Casey E, Sneag DB, Tan ET, et al. Lumbar spine bone MRI: a radiation-free approach utilizing CT-like imaging for diagnosing pars defects in the adolescent spine. AJNR Am J Neuroradiol. 2025. 10.3174/ajnr.A8852. 40935662 PMC12633688

[14] Abel F, Lebl DR, Gorgy G, Dalton D, Chazen JL, Lim E, et al. Deep-learning reconstructed lumbar spine 3D MRI for surgical planning: pedicle screw placement and geometric measurements compared to CT. Eur Spine J. 2024;33:4144–54. 38472429 10.1007/s00586-023-08123-3

[15] Kessler H, Clara M, Gillet R, Douis N, Ambarki K, Boubaker F, et al. Comparison of short TE CT-like MRI sequences and UHR-CT in cadaveric wrist study. Eur J Radiol. 2025;186:112032. 40068443 10.1016/j.ejrad.2025.112032

[16] Rai P, Janu AK, Shetty N, Kulkarni S. Current landscape of short-T2 imaging techniques in the musculoskeletal system: the past, present and future. J Magn Reson Imaging. 2025;62:969–85. 40256819 10.1002/jmri.29776 PMC12435157

[17] Hou B, Liu C, Li Y, Xiong Y, Wang J, Zhang P, et al. Evaluation of the degenerative lumbar osseous morphology using zero echo time magnetic resonance imaging (ZTE-MRI). Eur Spine J. 2022;31:792–800. 35015138 10.1007/s00586-021-07099-2

[18] Kaniewska M, Kuhn D, Deininger-Czermak E, Wolharn L, Finkenstaedt T, Guggenberger R. 3D zero-echo time and 3D T1-weighted gradient-echo MRI sequences as an alternative to CT for the evaluation of the lumbar facet joints and lumbosacral transitional vertebrae. Acta Radiol. 2023;64:2137–44. 37070233 10.1177/02841851231165487

[19] Website. Available: Lebel et al. Performance characterization of a novel deep-learning based MR image reconstruction pipeline. Accessed 02.07.2026. https://arxiv.org/abs/2008.06559v1

[20] Sneag DB, Queler SC, Campbell G, Colucci PG, Lin J, Lin Y, et al. Optimized 3D brachial plexus MR neurography using deep learning reconstruction. Skeletal Radiol. 2024;53:779–89. 37914895 10.1007/s00256-023-04484-4

[21] Fortin JD, Wheeler MT. Imaging in lumbar spinal stenosis. Pain Physician. 2004;7:133–9. 16868627

[22] Gamanagatti S, Rathinam D, Rangarajan K, Kumar A, Farooque K, Sharma V. Imaging evaluation of traumatic thoracolumbar spine injuries: radiological review. World J Radiol. 2015;7:253–65. 26435776 10.4329/wjr.v7.i9.253 PMC4585949

[23] Yi J, Hahn S, Lee H-J, Lee S, Park S, Lee J, et al. Deep learning reconstruction of zero echo time magnetic resonance imaging: diagnostic performance in axial spondyloarthritis. Eur Radiol. 2025. 10.1007/s00330-025-11843-3. 40707731

[24] Carretero-Gómez L, Fung M, Wiesinger F, Carl M, McKinnon G, de Arcos J, et al. Deep learning-enhanced zero echo time MRI for glenohumeral assessment in shoulder instability: a comparative study with CT. Skeletal Radiol. 2025;54:1263–73. 39572485 10.1007/s00256-024-04830-0 PMC12000158

[25] Lee C, Lee J, Mandava S, Fung M, Choi YJ, Jeon KJ, et al. Deep learning image enhancement for confident diagnosis of TMJ osteoarthritis in zero-TE MR imaging. Dentomaxillofac Radiol. 2025;54:302–6. 39989448 10.1093/dmfr/twae063 PMC12038245

[26] Breighner RE, Potter HG. CT-like contrast for bone imaging with ZTE-MRI. In: MRI of short and ultrashort-T_2 tissues. Cham: Springer International Publishing; 2023. p. 549–59.

[27] Ljungberg E, Damestani NL, Wood TC, Lythgoe DJ, Zelaya F, Williams SCR, Solana AB, Barker GJ, Wiesinger F. Silent zero TE MR neuroimaging: Current state-of-the-art and future directions. Prog Nucl Magn Reson Spectrosc. 2021;123:73–93. 10.1016/j.pnmrs.2021.03.002. 34078538 PMC7616227

